# Ontogeny of sexual size dimorphism revisited: Females grow for a longer time and also faster

**DOI:** 10.1371/journal.pone.0215317

**Published:** 2019-04-23

**Authors:** Virve Sõber, Siiri-Lii Sandre, Toomas Esperk, Tiit Teder, Toomas Tammaru

**Affiliations:** 1 Department of Zoology, Institute of Ecology and Earth Sciences, University of Tartu, Tartu, Estonia; 2 Faculty of Environmental Sciences, Czech University of Life Sciences Prague, Prague, Czech Republic; National Institute of Biology, SLOVENIA

## Abstract

Sex-specific mechanisms of the determination of insect body sizes are insufficiently understood. Here we use the common heath moth, *Ematurga atomaria* (Lepidoptera: Geometridae) to examine how larval growth trajectories differ between males and females. We monitored the development of 1379 larvae in controlled laboratory conditions. Sexually dimorphic development times during the first four instars were associated with sexual size dimorphism (SSD) in the beginning of the fifth (last) instar, when females were on average 15% heavier than males. Similarly, the duration of the last instar was about 13% longer in females. Further, we specifically focussed on the estimates of differential (instantaneous) growth rates of the larvae based on 24h mass increments of the 2^nd^, 3^rd^, 4^th^ and 5^th^ day in the beginning of the last instar. We calculated ‘allometric’ differential growth rates as the per-day increase in cube-root-transformed mass of the larvae. We found that allometric growth rates were slightly but significantly larger in females than in males. As this measure of growth rate (in contrast to the relative growth rate, based on the ratio of masses recorded at consecutive measurements) did not depend on body size, it allows an unambiguous separation of the effects of sex and size. We conclude that in accordance with an emerging general pattern, larger female body size in *E*. *atomaria* is achieved primarily by means of a longer growth period. Furthermore, our study shows that the differential growth rate can also be sexually dimorphic and contribute to SSD. This contribution, however, is lower than that of the development time by an order of magnitude. In addition to development periods and growth rates, other parameters of the non-linear growth curves of insect larvae also need to be considered in the context of SSD determination. In particular, weight loss prior to pupation was shown to be considerably larger in females than in males.

## Introduction

Sexual differences in body size (sexual size dimorphism, SSD) are a widespread phenomenon in most animal groups [1, 2]. Females are the larger sex in most invertebrates [3, 4, 5] and poikilothermic vertebrates [6, 7], whereas male-biased SSD is typical of birds [8, 9] and mammals [10, 11]. Evolutionary explanations of sexual size dimorphism primarily rely on selective forces operating in the adult stage. Fecundity selection is generally considered to cause female-biased SSD [4, 12, 13], but see [14], whereas male-biased size dimorphism is explained by sexual selection [1, 15, 16] but see [17]. Importantly, however, also the non-reproductive life stages may have a contribution. For example, sexual dimorphism may be affected by natural selection operating during the juvenile development. Such selective forces on growth schedules per se must depend on the proximate patterns and mechanisms of juvenile growth [18]. Ontogenetic mechanisms leading to sex-related differences in body size remain, however, insufficiently understood, which may result in an incomplete understanding of the selective factors that have shaped SSD (for insects, see however [19, 20].

In insects, there are various ontogenetic mechanisms which can lead or contribute to sexual size dimorphism. Either can the larger sex be larger from the beginning (implying sex-specific egg size that is infrequently examined, e.g. [21], have more larval instars (reviewed in [22]), or display more limited weight loss during metamorphosis [23, 24, 25]. Most research, however, has been focused on the question whether the larger size in one of the sexes is primarily achieved through longer developmental periods, or through faster growth of the juveniles. In insects, the accumulating evidence shows that the larvae of the larger sex tend to grow for a longer time than those of the smaller sex [26, 27, 28, 29]. However, sexually dimorphic growth rates have been reported as well [25, 30, 31, 32].

In most studies on sex-specific growth rates, the description of larval development is based on integral measures of juvenile growth [29, 30, 33]. We define an integral measure of growth rate as a measure which is calculated over entire developmental phases, most typically dividing final (adult) weights by development times expressed either as the duration of the larval period, or that of the entire immature period. Since insect larvae do not grow continuously, integral measures cannot reveal the proximate nature of sex-related differences in larval development [25, 34]. In particular, the growth curve of an insect larva has a complex shape due to distinct larval instars [35, 36, 37]. For this reason, a sex difference in an integral measure of growth rate may not result from an actually faster weight gain of a growing larva but, for example, may reflect a shorter ‘waiting time’ preceding a larval moult [38].

The growth curve can be described more adequately by using differential (or instantaneous) measures of growth rate [20, 25, 27, 37, 39]. Such measures rely on recording short-term mass increments at specific points of larval development, and are meant to approximate the derivative of the growth curve with respect to time (discussed in [34]). Estimating sexual differences in differential growth rates requires collecting data on larval growth trajectories through continuous monitoring, with special attention being paid to the ontogenetic phase of the larvae. Few examples of such approach exist. In a previous study, we compared differential growth rates of the two sexes in six lepidopteran species, and found differential growth rates to be merely marginally higher in females in the penultimate but not in the last instar [27]. Similarly, Stillwell and Davidowitz [26] reported inconclusive sex-related difference in differential growth rates in the sphingid moth *Manduca sexta*. A recent work on scarab beetles [25] found no evidence for the male-biased SSD, characteristic of this species, being related to sex differences in differential growth rates. This was inferred from the analysis of asymptotic growth functions fitted to empirical data.

The scarce and inconclusive evidence on sexually dimorphic growth rates as the proximate source of SSD in insects calls for additional case studies. As previous experience indicates that sex differences in growth rates–if any–tend to be minor, large sample sizes are required to obtain sufficient statistical power. Here, we used an unprecedented sample size to study sexual differences in growth curves in a moth with sexual size dimorphism, aiming at evaluating the potential of differential growth rate to the formation of the SSD. We compared the role of growth rate to that of sex-specific developmental period, and some other parameters of the growth curve. We discuss the results within the framework of evolutionary ecology of insect body size.

## Materials and methods

### Study species

The common heath moth, *Ematurga atomaria L*. (Lepidoptera, Geometridae, Ennominae), is a widespread day-flying lepidopteran abundant in various habitats of temperate Eurasia. The nectar-feeding adults are sexually dimorphic in size: pupal mass ratio, females to males is 1.16 (this study); resulting in SDI = (female size–male size)– 1 = 0.16 [40]. SSD of this magnitude is common, though not extreme, in Lepidoptera [3]. Selecting a study species with just a modest SSD ensured that sex-related differences are not a result of the corresponding differences in instar number. Namely, sex-related difference in instar number is a specific phenomenon, characteristic of species with high female-biased SSD [22], which is not the focus of the present study.

The wing span of the adults is 25–35 mm in males and 22–30 mm in females [41]. The species is univoltine in northern latitudes (including the study area), with the pupa as the overwintering stage [41]. Larval development of both sexes invariably consists of five instars ([41, 42, 43]; this study). The highly polyphagous larvae are external solitary feeders on leaves of their host plants. Host plants used in this study, common heather *Calluna vulgaris* L. and bilberry *Vaccinium myrtillus* L. (both Ericaceae), are dwarf shrubs abundant on moors and in woodlands in Northern Europe. Both species are common hosts of *E*. *atomaria* [44].

### Experimental design

To quantitatively compare the larval growth schedules of different sexes, we reared the larvae in standardised conditions in the lab. In 2009, the F1 offspring of the 75 field collected females were mated to the males from the same population to produce the F2 generation. 13 males were mated to one female each, and 48 males to two females each. This resulted in a total of 109 broods (offspring of a particular female). The offspring of the resulting half-sib and full-sib families were divided between two host plants: 12 larvae from each brood were reared on bilberry and three on heather. Using two different host plants was motivated in the context of other uses of the data set [43, 45, 46]. The larvae (N = 1379 reaching pupation) were reared individually in transparent 50 ml plastic vials at 22°C, exposed to a light/dark cycle of 16L:8D, and being provided with food *ad libitum*. Food plant sections were renewed every three days. The vials were arranged randomly on rearing trays with respect to brood and host plant. During the last larval instar, mortality of the insects was low (ca 1.4% per instar) and consistent across the two host plants.

We recorded development time of the larvae from hatching until the end of their fourth (penultimate) instar. The larvae were first weighed at the end of their fourth instar just prior to their last moult (during the intermolt growth stasis, recognised by morphological characters typical of this stage; [36]), this record is treated as the initial mass of last instar in the analyses. In the course of the 5th (last) instar, the larvae were weighed daily until cessation of feeding (the beginning of the wandering stage; e.g. [47]). This allowed us to record the maximal larval mass, and the day on which it was achieved. Due to technical difficulties associated with handling smaller larvae, such detailed measurements could be performed on the larvae of the last instar only. Larval period was considered to have ended when the larvae buried themselves into the substrate for pupation. The pupae were weighed and sexed a week after pupation.

### Variables and analyses

**Fig 1:**
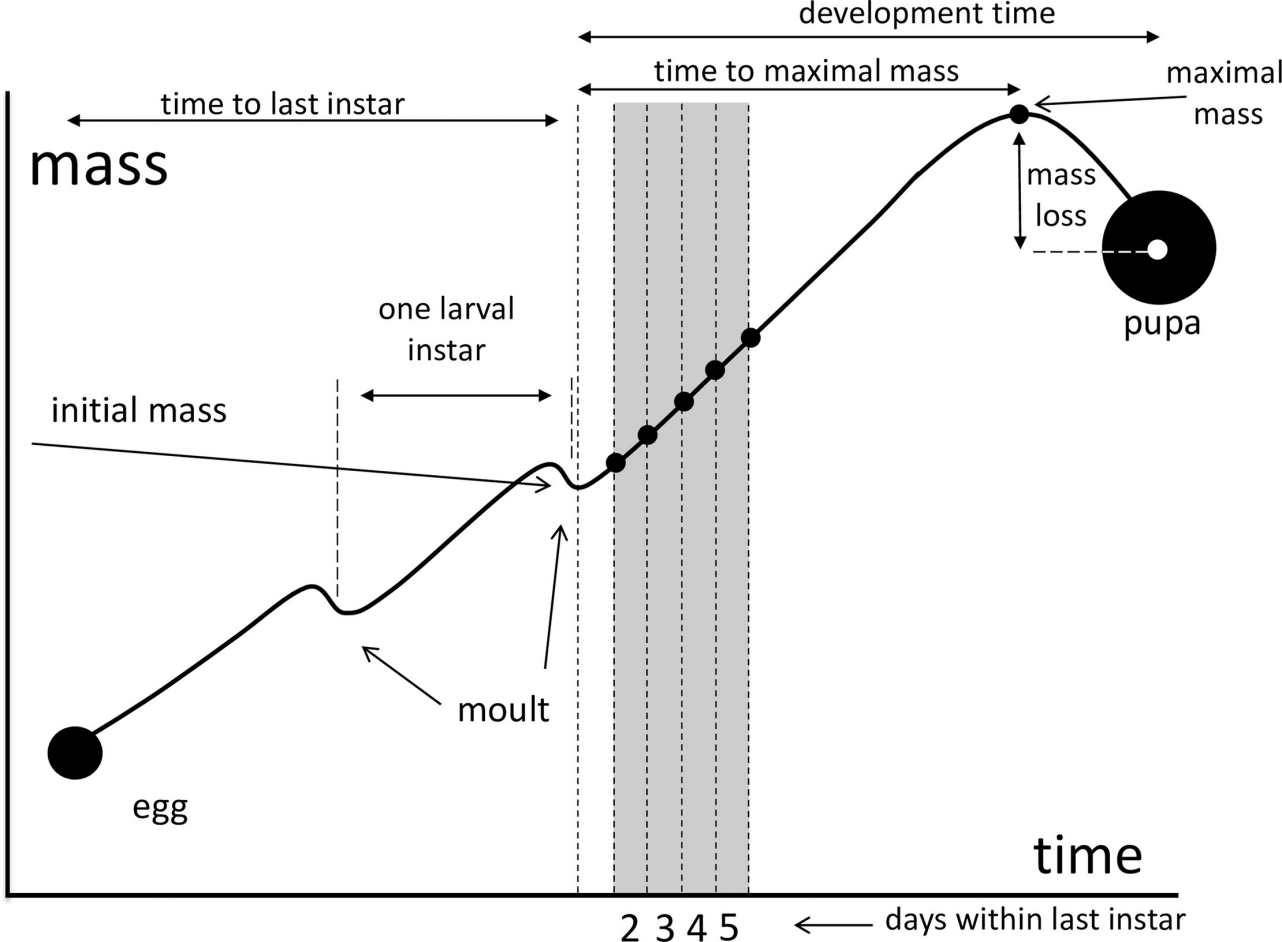
Growth trajectory of an insect larva, with an indication of descriptive statistics recorded in the present study. The grey bars represent the days in the beginning of the last instar, for which the measures of differential of growth rate were calculated. The presentation on the growth trajectory is schematic and bears no quantitative correspondence with that of *Ematurga atomaria* which has 5 larval instars.

To characterize larval development, various descriptive statistics of the last instar growth curve were recorded separately for the two sexes (Fig 1; Table 1). Significance tests for the sex-related differences in these characteristics were based on mixed ANOVAs with host plant as a fixed factor, and brood as a random effect. Analysing the data separately by host plants did not lead to any qualitatively different results.

**Table 1:** Growth parameters of the last (5th) larval instar (mean values±SE) of *Ematurga atomaria*, a lepidopteran with sexual dimorphism in pupal masses.

Variable	Female	Male	F_1;1243_	P	R^2^
Time to last instar (days)	18.17±0.070	17.58±0.065	36.95	<0.0001	0.071
Initial mass (mg)	29.18±0.15	25.30±0.11	475.65	<0.0001	0.25
Maximal mass (mg)	120.24±0.52	98.91±0.36	1503.01	<0.0001	0.37
Pupal mass (mg)	73.57±0.37	63.28±0.24	770.10	<0.0001	0.32
Development time (days)	11.46±0.066	10.10±0.059	335.23	<0.0001	0.18
Pupal mass/initial mass	2.55±0.014	2.52±0.011	3.35	0.068	0.15
Maximal mass/ initial mass	4.16±0.021	3.94±0.015	94.69	<0.0001	0.14
Maximal mass/ final mass	1.39±0.0038	1.37± 0.0032	38.10	<0.00011	0.051
Time to maximal mass (days)	9.75±0.058	8.67±0.052	247.77	<0.0001	0.18
Absolute mass increment, 2^nd^ day (mg)	9.89±0.17	8.76±0.14	24.46	<0.0001	0.002
Absolute mass increment, 3r^d^ day (mg)	11.84±0.19	10.43±0.17	33.06	<0.0001	0.007
Absolute mass increment,4^th^ day (mg)	12.58±0.22	11.37±0.19	17.99	<0.0001	0.0056
Absolute mass increment, 5^th^ day (mg)	12.03±0.23	10.68±0.21	21.83	<0.0001	0.0034
Absolute mass increment, 6^th^ day (mg)	12.61±0.25	10.92±0.22	0.98	0.322	0.0081
Absolute mass increment, 7^th^ day (mg)	11.19±0.26	9.05±0.25	8.40	0.003	0.0037
Absolute mass increment, 8^th^ day (mg)	8.68±0.26	5.14±0.28	17.28	<0.0001	0.013
Absolute mass increment, 9^th^ day (mg)	4.13±0.33	-1.65±0.38	135.96	<0.0001	0.045

Presenting daily absolute mass increments illustrates how growth slows down when the larva approaches pupation, this happens earlier in males than in females. Sexes are compared using mixed analysis of variance with food plant as an additional fixed factor and brood (offspring of an individual female) as a random factor, type III sum of squares. Effect size of sex is visualised by presenting factor-specific R^2^ values. Analysing the data separately by host plants did not lead to qualitatively different results.

Our day-specific estimates of differential (= instantaneous) growth rates are based on individual 24h mass increments of the 2^nd^, 3^rd^, 4^th^ and 5^th^ day of the last instar, covering about 37% of the duration of the instar. This time interval was chosen because, during this period in the beginning of the instar, the growth is affected by neither the preceding nor the subsequent moult (the ‘free growth’ period; [36]). This allowed us to focus on the process of actual mass accumulation. First day of the last instar was not considered because mass gain of that period is dominated by filling the gut rather than actual somatic growth (larvae moult with their guts being empty). During the second half of the last instar, larvae prepare for pupation, which involves slowing down growth and, finally, losing mass during the wandering stage [36, 38].

There are different ways of calculating differential growth rates based on the recorded 24h mass increments. Our aim was to identify an index of differential growth rate which shows least dependence on size, or, in other words, to choose a transformation which linearizes the growth curves of the larvae. This was essential in the context of comparing the growth of male and female larvae as those differ in average masses (Table 1). Using a size-independent index enabled us to unambiguously ascribe any sex-related differences to sex of the larva as such, and not to the different average sizes of the larvae representing the two sexes.

**Fig 2:**
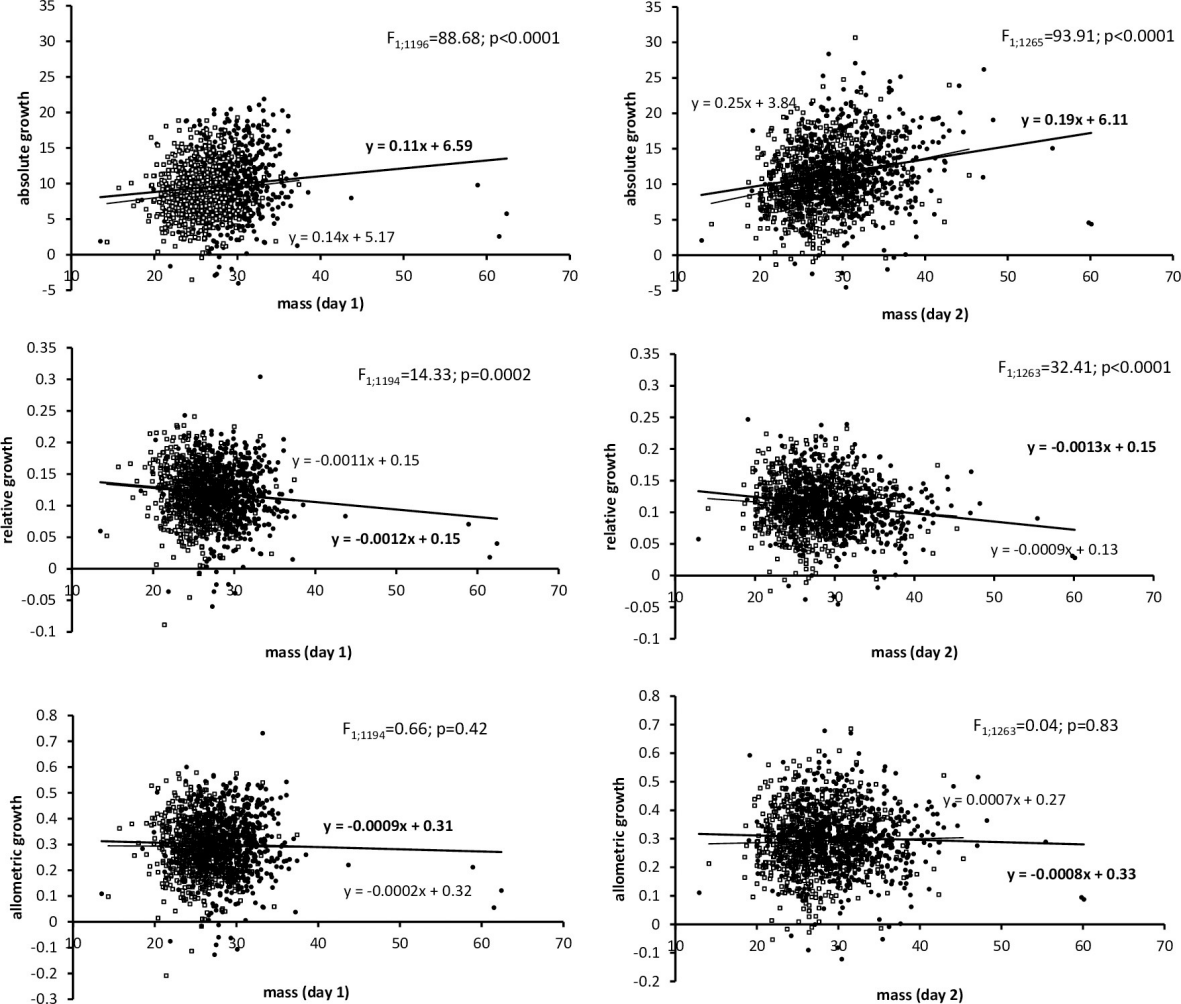
Size-dependence (results of mixed ANOVAs with sex and food plant as fixed factors and brood as a random factor) of three different measures of differential growth rates (absolute–mg·day^-1^; relative–day^-1^; allometric–mg^1/3^·day^-1^; see text) in female (solid circles) and male (empty squares) *Ematurga atomaria* larvae. Vertical axes represent growth rates calculated for the 2nd (left panels) and 3rd (right panels) days of larval growth in the final larval instar and horizontal axes represent masses (mg) recorded one day before the growth rate measurements. This way we avoided the situation that the two variables being correlated contain common elements, a situation known to cause statistical artefacts [49]. Regression lines and equations in bold represent females.

Quite obviously, absolute mass increments during a 24h period (mg/day) can be expected to correlate positively with larval body size. To eliminate size-dependence, we focussed on two different options how to express growth rate, based on masses recorded in the beginning (the 1st measurement) and at the end (the 2nd measurement) of the 24 h recording period. First, the differential growth rates were calculated as [the cubic root of mass at 2nd measurement–the cubic root of mass at 1st measurement], following the observation of Tammaru and Esperk [37] that growth of a lepidopteran larva usually follows a cubic function during the period of free growth. This measure was here termed the *allometric* differential growth rate (see S1 File for a more detailed explanation). Second, we employed the method relying on the assumption of exponential growth [48]. Accordingly, the *relative* differential growth rates were calculated as [log_10_ (mass at 2nd measurement / mass at 1st measurement)]. To evaluate the size-independence of both the allometric and relative differential growth rates, the values of these two indices for the 2nd and 3rd day of the last instar (most certainly representing the free growth period) were regressed on the values of larval body size (Fig 2).

Next, we tested for sex-related differences in the differential growth rates, both the allometric and relative differential growth rates. The values of the differential growth rate for the 2^nd^, 3^rd^, 4^th^, and 5^th^ days of the last instar were treated in the analysis as repeated measurements on each individual. These values were compared between the two sexes using mixed analysis of variance, with host plant, sex, day and sex*day as fixed factors; brood and ID of an individual were treated as random factors. The interaction term was included to test whether we succeeded to analyse the free growth period of the larvae (see below for discussion). Denominator degrees of freedom were derived from the number of larvae (and not from that of the measurements, in order to avoid pseudoreplication). The mixed analyses of variance were run in SAS 9.4 (PROC MIXED; [50, 51]. Sex-specific R^2^-s were obtained using the function rsquaredGLMM in the MuMIn package [52] in R 3.4.3 [53].

## Results

Sex-related differences in growth patterns were present both before and during the last (5^th^) instar of *E*. *atomaria* larvae. Female-biased SSD could be observed already in the beginning of the last instar (Table 1): on average, the female larvae were about 1.15 times the mass of the male larvae at that time. The higher initial mass of females at the beginning of 5^th^ instar was coupled with growing for a longer time during the first four instars: for females, it took on average a day (4.5%) longer to grow from hatching from the egg until the beginning of the 5^th^ instar (Table 1); egg size is not sexually dimorphic in *E*. *atomaria* (M. Martverk, unpublished).

**Fig 3:**
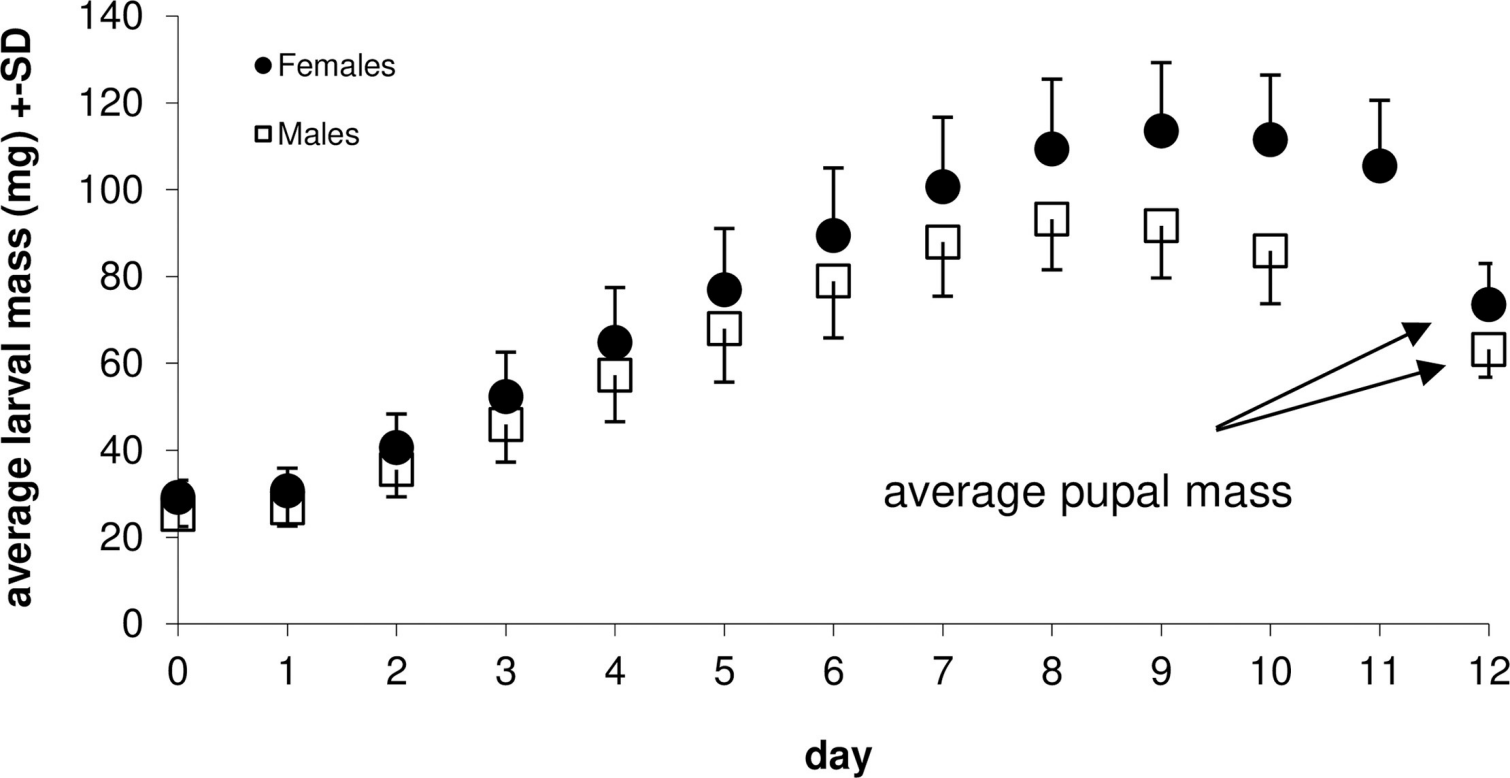
Average larval masses of the two sexes of *Ematurga atomaria*, presented from the beginning of the 5th instar until the median duration of the instar (11 days in females and 10 days in males). Average pupal mass denotes average pupal mass of females and males, recorded a week after pupation.

Female insects stayed larger throughout the last instar until pupation (Fig 3): both their maximal masses and pupal masses were larger, on average, compared to males (Table 1). SSD (female mass: male mass) of the last instar was 1.22 in maximal masses and 1.16 in pupal masses. The females gained more mass during the last instar, but they also lost more mass between reaching the maximal mass and pupation (note the sexually dimorphic maximal mass/ initial mass and maximal mass/ final mass ratios, Table 1). The larger maximal masses in the females during the last instar were associated with longer (0.9 days on average, or 12.5%) growing time up to the point when the maximal mass was achieved (Table 1). The duration of the entire last instar was longer (by 13.4%, or one day, on average; Table 1) in females than in males. These patterns were highly consistent between the two host plants used, and we therefore do not present the results separately by host plants.

Sex-related differences in the measures of differential larval growth rate depended on the way how the growth rates were calculated. We detected sex-specific differences in the allometric differential growth rates: females grew slightly faster than males: 0.279 mg^1/3^day^-1^ vs. 0.270 mg^1/3^day^-1^. In contrast, the relative growth rates (logarithm of relative increase per day, see above) showed no sex-specific differences. In particular, during the period from 2^nd^ to 5^th^ day of the last instar, the female larvae grew on average 1.264 times heavier within 24h, whereas the males grew 1.267 times. Importantly, the sex*day interaction did not attain significance in either of the analyses with differential growth rates (Table 2). The absence of the interaction indicates that we succeeded in analysing the period of ‘free’ larval growth, which is unaffected by preparations to pupation. As growth slows down earlier in males than in females (Table 1, Fig 3), including the slowing down period into our analysis would have been reflected in a significant sex*day interaction. Capturing the free growth period is further confirmed by the qualitatively consistent results of an alternative analysis in which we conservatively excluded the 5^th^ day from the analysed period (results not shown). Moreover, qualitatively consistent patterns (females growing faster in terms of the allometric differential growth rate) were also observed in all cases when the 2^nd^ to 5^th^ days of growth were analysed separately, but only on the 3^rd^ day was the sexual difference statistically significant (F_1;1265_ = 9.75; p = 0.0018).

**Table 2:** The results of mixed ANOVAs for three different measures of differential growth rate of the last instar larvae of *Ematurga atomaria*.

	Effect	NomDF	Ddf	F	P
Absolute increments*	Sex	1	1271	18.96	<0.0001
	Day	1	4135	37.75	<0.0001
	Plant	1	1271	4.45	0.035
	Sex:day	1	4135	0.013	0.91
	Sex:plant	1	1271	4.51	0.033
Allometric***	Sex	1	1271	11.08	0.0009
	Day	1	4135	202.58	< .0.0001
	Plant	1	1271	80.41	< .0.0001
	Sex:day	1	4135	0.51	0.47
	Sex:plant	1	1271	2.39	0.12
Relative**	Sex	1	1271	0.124	0.72
	Day	1	4135	847.11	< .0.0001
	Plant	1	1271	68.26	< .0.0001
	Sex:day	1	4135	0.31	0.58
	Sex:plant	1	1271	1.58	0.21

The mass increments of 2^nd^, 3^rd^, 4^th^ and 5^th^ days within the last instar were treated as repeated measurements on particular individuals. Host plant, sex, day and sex*day were included into the model as fixed factors, and brood (offspring of an individual female) and individual larva (nested in brood) as random factors; type III sum of squares. Removing non-significant interactions from the models did not have a qualitative effect on other statistics.

* Absolute increments represent individual 24h mass increments.

** Relative differential growth rates were calculated as [log_10_ (mass at the 2^nd^ measurement / mass at the 1^st^ measurement)].

*** Allometric differential growth rates were calculated [the cubic root of mass at 2nd measurement–the cubic root of mass at 1st measurement].

The analysis of size-dependence of the different measures of differential growth rate (Fig 2) revealed that the allometric growth rate shows no dependence on larval body size. The cubic-root transformation of body size appeared thus to be appropriate in linearizing larval growth trajectories. In contrast, the relative growth rates were lower in larger larvae.

## Discussion

In the studied moth, sexual size dimorphism (SSD) appears to be associated, along with other mechanisms discussed below, with the longer growing time of the larger sex. The average body mass of the female larvae, being coupled with longer development periods over the first 4 instars, was higher than that of the male larvae by the end of the penultimate instar. Females grew for a longer time than males also during their last instar. The fact that SSD did not increase in the course of the last instar (the females were about 1.16 times heavier both in the beginning of the last instar, and as pupae) could be interpreted as questioning the *causal* connection between longer development time and larger sizes. However, the positive association between mass gain and development time was observed also when the period from the beginning of the last instar until achieving mass maximum (higher in females) was considered.

Longer development time of the larger sex seems to be a common feature among different insect groups [25, 27, 28, 29, 32]. Our results also add to the growing body of evidence showing that SSD appears already during an early larval stage in insect species with no sex-specific difference in the number of instars [20, 24, 25, 27]. However, the species in which the number of instars varies between sexes appear to be different in this respect. In such insects, SSD can be attributed solely to different growth patterns in the last instar [22]. More generally, differences in development time have been observed also in the formation of adaptive size differences other than between sexes. In particular, recent evidence shows that size differences among populations [34, 46] and seasonal generations [38] in Lepidoptera also arise in the same way, i.e. through longer growth periods of the ultimately larger individuals.

Additionally, however, differential growth rates were slightly but significantly higher in female larvae. This difference was detected using a measure that unambiguously separated the effects of sex and body size (the allometric differential growth rate, i.e. the increase in cube-root transformed body mass). Indeed, an unambiguous test of sex-related differences in growth rates should rely on a measure of growth rate that does not depend on size. Here we showed that, in consistence with Tammaru and Esperk [37], the increase in cube-root transformed mass per unit of time (the allometric growth rate) meets this requirement.

An alternative measure of differential growth rate–the relative differential growth rate–did not depend on the sex of the larva. The relative growth rates, were, however, also found to depend negatively on body size (in accordance with [37]). This implies that, in terms of the relative growth rate, the positive effect of female sex *per se* was compensated precisely by the negative effect of the larger size of female larvae. Different results found for the different indices of growth rate demonstrate that caution is needed when choosing the measure of growth rate, and interpreting the results. Moreover, our study shows that considerable sample sizes are needed to obtain reliable results, due to just minute sexual differences in differential growth rate and high residual variance in this variable. The sample size of the present study (1379 larvae) can be considered large in comparison to that of most analogous studies, which may partly explain why we were able to demonstrate differences in differential growth rates in this but not in other similar experiments [27, 34, 38].

The detected sexual difference in instantaneous growth rates does not challenge the overall conclusion that larger body size in insects is primarily achieved via longer growth periods and not via higher growth rates. A simple calculation (S 2) shows that the detected sex-related difference in the allometric differential growth rate could lead to a 1.03-fold relative mass difference when present during five days of growth. At the same time, prolonging the free growth period by one day (6 instead of 5) would make the females 1.22 times heavier as compared to males. The pattern of merely minor sex-related differences in differential growth rate suggests that a major (evolutionary or plastic) change in this parameter should be disadvantageous [27] and generally ‘avoided’. Indeed, higher growth rates are known to incur various costs such as higher predation risk (e.g., [54, 55], higher mass loss at metamorphosis [56], higher metabolic rate and reduced investment in energy storage [57], oxidative stress [58] and impaired immune function [59]. Accordingly, most examples of plasticity in growth rates in insects can perhaps be understood not as condition-dependent acceleration of growth but rather as slowing down growth (‘killing time’) in situations in which it is adaptive not to enter a certain developmental stage too early [60, 61, 62, 63].

Our results also showed that considering only development time and growth rate is not sufficient to describe fully sexual differences in growth patterns, even within just one larval instar. In particular, there was a negligible difference in SSD values in the beginning of the last instar and in the pupal stage of *E*. *atomaria*. Nevertheless, the sex difference in peak masses of the larvae was considerably larger, implying that female larvae both gained and thereafter lost more mass than males, both in absolute and relative terms. Previous work has associated the mass loss between the cessation of growth and pupation with the energy cost of the wandering stage before pupation, and with physiological preparations for overwintering [38, 39]. Irrespective of both proximate and ultimate reasons behind the phenomenon, it is however clear that differences in the weight loss at this stage may substantially contribute to the patterns of body size (see [38] for size differences among seasonal generations). This is analogous to the frequently sexually dimorphic mass loss upon adult eclosion [23, 24, 64]. The patterns of sex-specific weight loss emphasize the need of considering the non-linear character of larval growth curve. This confirms the message that integral measures of larval growth are oversimplified and therefore of limited use in contexts in which the focus is on proximate physiological mechanisms [34] (see S 3 for the reanalysis of the data of the present article using integral measures).

In conclusion, we show that sexual size dimorphism in *E*. *atomaria*, being present already in the early stage of larval development, mainly results from prolonged larval growth of the females. This implies that increased development time (and, consequently, higher juvenile mortality) may form the primary cost of achieving large female size. However, the present study may be the first on insects to show that the differential growth rate can also be (slightly) higher in females, and that it has the potential to contribute to the formation of SSD. Our study is nevertheless consistent with the idea that differential growth rates are relatively conserved in insects and tend not to respond readily to selection pressures; size differences are primarily formed by other means when ‘needed’ (see [30] for a similar conclusion about vertebrate animals). This may be an indication of high developmental and/or physiological costs of increasing growth rate. As a methodological contribution, this study shows that the measures of growth rate should be chosen carefully in empirical studies. In addition, parameters other than development time and growth rate should be considered while studying the mechanisms of formation of adaptive size differences.

## Supporting information

S1 Data The recordings of development time (days) and body mass (mg) of the *Ematurga atomaria* larvae (N = 1379) during their last, 5^th^ instar. (TXT)

S1 File The absolute, relative and allometric growth rates, and the rationale behind them. (DOCX)

S2 File Comparing the contributions of 1) sexually dimorphic instantaneous growth rate, and 2) longer development periods of females to the formation of SSD. (DOCX)

S3 File Reanalysis of the data of the present article using integral measures of growth rate. (DOCX)

S1 Table Three different integral measures of growth rates of the last (5th) larval instar (mean values±SE) of Ematurga atomaria, a lepidopteran with sexual dimorphism in pupal masses. (DOCX)
