# Supplementary material for: Ontogeny of sexual size dimorphism revisited: Females grow for a longer time and also faster
Source: PLoS One. 2019 Apr 23;14(4):e0215317. doi: 10.1371/journal.pone.0215317 (PMC6478289; doi:10.1371/journal.pone.0215317)
Supplement: S1 File — (DOCX) [file pone.0215317.s002.docx]

**Supplement 1.**

**The absolute, relative and allometric growth rates, and the rationale behind them**

Both allometric and relative differential growth rates are calculated from two consecutive measures of larval mass taken 24h apart in time. The difference between allometric and relative measures is just in the mathematical transformation applied. The allometric growth rate measures the rate of increase in the cube-root transformed mass of the larvae (the term reflects the assumption that growth rate and mass are related to each other through a power function, which is consistent with the standard use of the term ‘allometry’ in biology). In turn, relative growth rate reflects a relative change in the mass of the larva (mass recorded at the second measurement divided by mass recorded at the first measurement).

The aim of comparing these two measures of differential growth rate was to find the measure which shows no size-dependence. As male and female larvae differ in average body mass, using a size-independent measure of growth rate would allow us to unambiguously separate the effects of body mass and sex. Allometric growth rate would be size-independent if the growth of the larva followed a cubic function. Relative growth rate would be size-independent if the growth of the larva followed an exponential function.

We found that allometric growth rate was size-independent whereas relative growth rate was not. This tells us that the growth rate of the larvae follows a cubic function rather than an exponential one, and suggests that the allometric growth rate should be used as a size-independent measure of larval growth rate.
