# Supplementary material for: Ontogeny of sexual size dimorphism revisited: Females grow for a longer time and also faster
Source: PLoS One. 2019 Apr 23;14(4):e0215317. doi: 10.1371/journal.pone.0215317 (PMC6478289; doi:10.1371/journal.pone.0215317)
Supplement: S2 File — (DOCX) [file pone.0215317.s003.docx]

**Supplement 2.**

**Comparing the contributions of 1) sexually dimorphic instantaneous growth rate, and 2) longer development periods of females to the formation of SSD**

It is assumed that square root of body mass increases linearly in time (allometric differential growth rate is size-independent, see text), all parameters are derived from the data of the current study.

1) Assume that a larva weighs 27 mg (or has cubic-root transformed mass of 3 mg^1/3^) at the beginning of its last larval instar. A female larva which grows at the rate 0.279 mg^1/3^d^-1^ for 5 days (the ‘free’ growth period, see text), attains cubic-root transformed mass of 3 + 5*0.279 = 4.395 mg^1/3^, or (if back-transformed) 84.89 mg.

A male larva with an equal initial mass which grows at the rate 0.270 mg^1/3^d^-1^ for 5 days, attains cubic-root transformed weight of 3 + 5*0.270 = 4.350 mg^1/3^, or 82.31 mg.

The assumed (and actually recorded in this study) difference in allometric differential growth rates will thus lead to SSD equal to 84.89/82.31 = 1.031.

2) Assume that, for the same initial mass, both larvae have growth rates equal to 0.2745 mg^1/3^d ^-1^ (the average across sexes) but males grow for 5 days whereas females grow for 6 days.

Males will attain masses: 3 + 5*0.2745 = 4.372 mg^1/3^, or 83.60 mg.

Females will attain masses: 3 + 6*0.2745 = 4.647 mg^1/3^, or 102.1 mg.

If females grow for one day longer than males, the resulting SSD is 102.1/83.60 = 1.22.
