# Supplementary material for: Ontogeny of sexual size dimorphism revisited: Females grow for a longer time and also faster
Source: PLoS One. 2019 Apr 23;14(4):e0215317. doi: 10.1371/journal.pone.0215317 (PMC6478289; doi:10.1371/journal.pone.0215317)
Supplement: S3 File — (DOCX) [file pone.0215317.s004.docx]

**Supplement 3.**

**Reanalysis of the data of the present article using integral measures of growth rate**

In addition to differential growth rates, integral growth rates of the larvae in their last instar were calculated on the basis of the initial masses of the last instar and pupal masses. In consistence with the way how we treated differential growth rates (see Methods), three different indices of integral growth rate were computed. The absolute integral growth rates were found as [(pupal mass - initial mass)/ number of days in the last instar], allometric integral growth rate was derived in the same way from the cube root transformed values of body mass, whereas the relative integral growth rate was calculated as [log10 (pupal mass/ initial mass)/ number of days in the last instar].

Interestingly, the effect of sex on integral growth rates was inconsistent with the effect of sex on differential growth rates. The absolute integral growth rates calculated over the entire duration of the last instar did not differ significantly between sexes. Unexpectedly, males could be concluded to grow faster than females, based on both the allometric integral growth rate and on the relative integral growth rate (see S3.Table below).
