# Supplementary material for: Ontogeny of sexual size dimorphism revisited: Females grow for a longer time and also faster
Source: PLoS One. 2019 Apr 23;14(4):e0215317. doi: 10.1371/journal.pone.0215317 (PMC6478289; doi:10.1371/journal.pone.0215317)
Supplement: S1 Table — (DOCX) [file pone.0215317.s005.docx]

**S3. Table** Three different integral measures of growth rates of the last (5^th^) larval instar (mean values±SE) of *Ematurga atomaria*, a lepidopteran with sexual dimorphism in pupal masses.

| Variable | Female | Male | F_1;1243_ | P | R^2^ |
| --- | --- | --- | --- | --- | --- |
| Absolute (mg/day) | 3.99±0.0044 | 3.87±0.0035 | 2.33 | 0.131 | 0.39 |
| Allometric ((mg⅓)/day) | 0.099±0.00097 | 0.11±0.00088 | 22.49 | <0.0001 | 0.39 |
| Relative | 0.036±0.00034 | 0.041±0.00033 | 65.45 | <0.0001 | 0.41 |

Sexes are compared using mixed analysis of variance with food plant as an additional fixed factor and brood (offspring of an individual female) as a random factor, type III sum of squares. Effect size of sex is visualised by presenting factor-specific R^2^ values. Analysing the data separately by host plants did not lead to qualitatively different results.
